# Healing of Extraction Sites after Alveolar Ridge Preservation Using Advanced Platelet-Rich Fibrin: A Retrospective Study

**DOI:** 10.3390/bioengineering11060566

**Published:** 2024-06-03

**Authors:** Antonia Samia Khaddour, Răzvan Eugen Ghiță, Mihaela Ionescu, Radu Gabriel Rîcă, Veronica Mercuț, Horia Octavian Manolea, Adrian Camen, Emma Cristina Drăghici, Andrei Radu, Sanda Mihaela Popescu

**Affiliations:** 1 Department of Oral Rehabilitation, University of Medicine and Pharmacy of Craiova, 200349 Craiova, Romania; antoniasamia11@gmail.com (A.S.K.); emma.draghici@umfcv.ro (E.C.D.); andrei.radu0692@gmail.com (A.R.); sanda.popescu@umfcv.ro (S.M.P.); 2 Department of Medical Informatics and Biostatistics, University of Medicine and Pharmacy of Craiova, 200349 Craiova, Romania; 3 Department of Dental Technology, University of Medicine and Pharmacy of Craiova, 200349 Craiova, Romania; radu.rica@umfcv.ro; 4 Department of Prosthetic Dentistry, University of Medicine and Pharmacy of Craiova, 200349 Craiova, Romania; veronica.mercut@umfcv.ro; 5 Department of Dental Materials, University of Medicine and Pharmacy of Craiova, 200349 Craiova, Romania; horia.manolea@umfcv.ro; 6 Department of Oral and Maxillofacial Surgery, University of Medicine and Pharmacy of Craiova, 200349 Craiova, Romania; adrian.camen@umfcv.ro

**Keywords:** alveolar ridge preservation, extraction sites, socket preservation, bone loss, advanced platelet-rich fibrin, cone beam computed tomography systems, horizontal changes, vertical changes

## Abstract

Background: Over time, numerous surgical procedures and biomaterials have been proposed for the reconstruction of post-extraction bone defects, each with their advantages and disadvantages. The main objective of this study was to evaluate dimensional changes in the alveolar bone 3 months after tooth extraction, before implant planning, comparing alveolar ridge preservation (ARP) with spontaneous healing. Methods: A total of 84 patients with non-restorable molars were included in the study. Forty-two patients received ARP with advanced platelet-rich fibrin (A-PRF) and spontaneous healing was evaluated in these patients. Cone beam computed tomography (CBCT) analysis performed before and after surgical intervention was used to determine the changes in vertical and horizontal bone dimensions produced after tooth extraction. Results: CBCT measurements showed reduction in both vertical and horizontal alveolar bone size in both groups. For the study group, the alveolar parameters (height, width) were higher compared to the control group. The percentage variations between dimensional differences from the two groups were 38.58% for height, and for width were 36.88% at 0 mm, 35.56% at 3 mm, 36.61% at 5 mm, and 38.73% at 7 mm. The differences were statistically significant (*p* ˂ 0.0005). Conclusions: The results obtained after ARP with A-PRF showed a reduced loss of bone volume compared to spontaneous healing.

## 1. Introduction

The size of the alveolar process is dependent on the presence of dental units [1], so the loss of teeth triggers a series of biological processes, affects the masticatory efficiency, and causes local anatomic changes [2,3] and structural changes in the periodontal tissues, ultimately leading to the atrophy of the alveolar ridge [3,4]. Implant restorations have become a common solution for replacing missing teeth. To achieve long-term success and to have considerable aesthetic results, it is necessary to have sufficient bone volume, both vertically and horizontally [5]. Post-extraction wound healing is a very important aspect, which can be defined as a complex and dynamic process of replacing devitalized and missing cellular structures and tissue layers [6,7]. Normal post-extraction alveolus healing consists of four essential phases: hemostasis, inflammation, proliferation, and the bone modeling and remodeling phase [8], which is associated with vertical and horizontal bone loss [9]. However, there are several factors that can influence these four phases [10]. These include systemic health, such as diabetes [11], smoking [12,13,14], radiation, antiresorptive drugs, and chemotherapy [15,16]. Any disruption of wound healing can lead to excessive bleeding or the absence of blood clots [17,18]. The extent of the dimensional change in bone and soft tissues is influenced by several systemic, local, and anatomical factors. Thus, the amount of newly formed bone and the extent of volumetric reduction in the alveolar ridge cannot be easily predicted and may vary not only between individuals but also between different extraction sockets in the same patient [19].

After tooth extraction, if the alveolus is left to heal spontaneously, without application of methods to preserve the alveolar ridge, then resorption and advanced bone atrophy occur [18]. During bone healing, alveolar walls will undergo the process of three-dimensional resorption, leading to significant morphological and topographical changes in hard and soft tissues and altering the outline of the pre-extractive alveolar process [19]. The post-extraction bone resorption with significant changes in the dimensions of the alveolar ridge depends on the type of tooth. The risks associated with this dimensional change are particularly evident in the anterior area, due to the presence of the thin bone bundle that may predispose to severe alveolar resorption [7,19,20]. Also, for the posterior area, among the most important local factors involved in alveolus healing is the anatomy of the alveolar process and the basal bone. Thus, the number of remaining alveolar walls after extraction, as well as their quality, is very important in the healing process of the post-extraction alveolus If the number of remaining alveolar walls is less, and even more so if they are thin, post-extraction alveolar healing will be impaired [21]. It is important to point out that the anatomy of molar post-extraction alveoli differs greatly from the anatomy of post-extraction alveoli in anterior areas, by their size, by the thickness of the oral and lingual cortices, and by the presence of septa [8]. In addition, there are differences between the maxillary and mandibular post-extraction areas because of the phenomenon of pneumatization of the maxillary sinus [22]. It has been reported that greater bone resorption occurs following spontaneous healing and this is especially greater in the molar areas [23]. There are situations when, if the alveolar ridge is not preserved, the post-extraction areas near the mandibular canal or the maxillary sinus suffer such a great resorption that can impair the placement of dental implants [8,24].

Alveolar ridge preservation (ARP) cannot stop the physiological resorption process, but it can significantly reduce it [8,25,26]. Therefore, in cases when immediate implantation is not possible, alveolar ridge preservation is recommended to reduce the decrease in the volume of the alveolar bone, in order to be able to withstand the dental implant insertion procedures [2,27]. The new bone formation and the alveolar ridge dimension preservation after extraction will facilitate the success of the implantoprosthetic treatment. Also, the preservation of the contour and volume of the soft tissues is necessary to obtain aesthetics and support of the implant by healthy peri-implant tissues [19]. Alveolar ridge preservation (ARP) involves any procedure that aims to limit the dimensional loss of the alveolar ridge after extraction, promote new bone formation in the post-extraction alveolus, preserve the contour of the alveolar ridge, and promote soft tissue healing [19].

After tooth extraction, bone resorption is most active in the first year and is most pronounced in the first three months, with 30–50% of the width [28,29,30,31,32] and 10–20% of the height of the alveolar bone being lost within 12 months of extraction [9,33]. Over time, several surgical procedures have been proposed to reduce the degree of bone resorption. Among them, flapless tooth extraction, alveolar ridge preservation, and immediate implant placement were mentioned [26]. In any situation, bone regeneration techniques are able to provide positive and lasting results [34].

In recent years, many variants of alveolar ridge preservation have been tried, one of them being the alveolar graft with a single biomaterial interposing a barrier element [3]. However, there has been no resolution regarding the best substitute material for alveolar preservation: autogenous, allogeneic, xenogeneic, or alloplastic graft [3,35,36]. The decision regarding the choice of the type of biomaterial differs depending on the case [3]. One must take into account mainly the results obtained and the loss in bone volume compared to spontaneous healing [35]. The chosen material must have predictable results and cost efficiency [3,35].

Alveolar ridge preservation is effective but technically delicate, requiring specific surgical skills [1,2,4,37]. ARP is usually performed immediately after tooth extraction or, rarely, it is postponed for a period of 6–8 weeks for reasons such as the presence of acute infections. A recent study reports that 6–8 weeks after tooth extraction, the alveolar ridge undergoes minimal dimensional changes [38]. In addition, the size of the soft tissues can be modified due to the physiological processes occurring after the loss of teeth [1].

Platelet concentrates were used to enhance post-extraction healing [39,40]. Platelet- rich fibrin (PRF), the most studied type of platelet concentrates for accelerating wound healing, especially for soft-tissue migration [16,41,42,43] is a platelet product rich in slow-release autogenous growth factors that improve healing. PRF is a completely autologous product prepared exclusively from the patient’s blood, with a rich network of fibrin, platelets, leukocytes, and cytokines [41,43]. By modifying the centrifugation process, different types of platelet concentrates can be obtained [40,44]. The advantages of using platelet-rich fibrin in post-extraction wounds are as follows: an improvement in soft tissue healing, reduction in inflammation and pain, as well as acceleration of bone formation [41,45,46,47,48,49,50].

A-PRF was obtained through low-speed centrifugation of the whole blood, without additives, which improved the distribution and quality of growth factors [39,40,51]. Unlike other platelet products, A-PRF has a more homogeneous distribution of the amount of platelets and leukocytes present [39] and it has a thicker, more reticulate network [40,52]. Most studies have reported improved soft- and hard-tissue regeneration when using A-PRF alone or with other grafting material [42,46,47,48]. The use of A-PRF ensures success in obtaining larger height dimensions and in maintaining favorable ridge profile, probing depth, and gingival margin level of adjacent teeth after extraction. A-PRF also increased bone density, vital bone, epithelial healing, and control of postoperative pain and swelling after tooth extraction and in the treatment of alveolar osteitis [46].

The aim of this study was to evaluate on cone beam computed tomography (CBCT) the bone changes produced after tooth extraction from the maxillary and mandibular molar areas by comparison of the study group with A-PRF alveolar ridge preservation to the control group with spontaneous healing.

## 2. Materials and Methods

### 2.1. Study Design

This retrospective cohort study complied with Strengthening the Reporting of Observational Studies in Epidemiology (STROBE) guidelines [53].

In the present study, the patients’ charts were analyzed, from which the demographic data of the patients, information related to the diagnosis, treatment plan, and complementary examinations, were extracted. CBCT analysis was performed in patients who had dental extractions in the areas of the maxillary and mandibular molars, in which either healing was achieved spontaneously, or they were treated by alveolar ridge preservation, according to the patient’s choice. All patients were selected from the Department of Oral Rehabilitation, University of Medicine and Pharmacy of Craiova, and treated between January 2022 and January 2023. The study protocol was approved by the Ethics Committee of the University of Medicine and Pharmacy of Craiova (Approval Number 230/28.11.2022). Surgical interventions were undertaken with the understanding and written informed consent of each subject. The patients were treated in full accordance with the applicable ethical principles, including the World Medical Association Declaration of Helsinki (version 2013).

### 2.2. Patients Selection

A group of 84 patients of both genders, non-smokers, without general systemic diseases, with maxillary and mandibular molars indicated for extraction, were included in the study. Depending on the patient’s request for the ARP procedure, the study group (42 patients) was formed, while in the control group (42 patients), the patients who chose simple extraction as the treatment option were included.

Inclusion criteria were as follows:

- Patients over 18 years of age.
- Maxillary or mandibular posterior teeth (first and second molars) that cannot be retained due to severe caries, chronic periapical periodontitis, failed endodontic treatments, or fatigue tooth fractures.
- At least 2 or more bone plates exist at the extraction site.
- Patients without serious systemic diseases (ASA I and ASA II).
- Good compliance.
- Patients who want implant restorations.

Exclusion criteria were as follows:

- Patients in whom the insertion of implants was performed immediately after the extraction.
- Severe hypertension, diabetes, kidney, and liver disease.
- Patients taking anticoagulants, systemic steroid treatment, or systemic bisphosphonates.
- Smokers (>10 cigarettes a day), alcoholics, drug users.
- Pregnant or lactating patients.
- Patients with previous radiation therapy in the surgical area.
- Bad oral hygiene.
- Acute infection of the tooth that will be extracted.

All dental charts included 2 CBCTs, one before the tooth extraction and another one made 3 months after the surgical procedure. CBCTs were recommended for the purpose of performing prosthetic implant treatment.

### 2.3. Treatment Procedures

#### 2.3.1. Preoperative Work-Up

Each patient was evaluated, and a dental chart was made. Preoperative examination included general condition assessment, clinical examination, CBCT analysis, photographs, routine blood tests, and sessions whereby the patients were instructed on how to correctly perform oral hygiene. After the diagnosis and the indication for extraction, the treatment plan was selected for each patient according to his wishes and it was accepted by each patient through written consent. For the study group, the treatment plan included anesthesia, extraction, and A-PRF making, and for the control group, anesthesia and simple extraction. For both groups, antibiotic therapy (1 g amoxicillin with clavulanic acid BID or 600 mg of clindamycin BID for patients allergic to penicillin) was prescribed starting one day prior to the procedure and continued for a total of 5 days. After 3 months, for both groups, a second CBCT evaluation was carried out with the aim of implantoprosthetic treatment. CBCT was taken to measure the height and width of alveolar bone before extraction.

#### 2.3.2. Preparation of A-PRF Membranes

**Figure 1:**
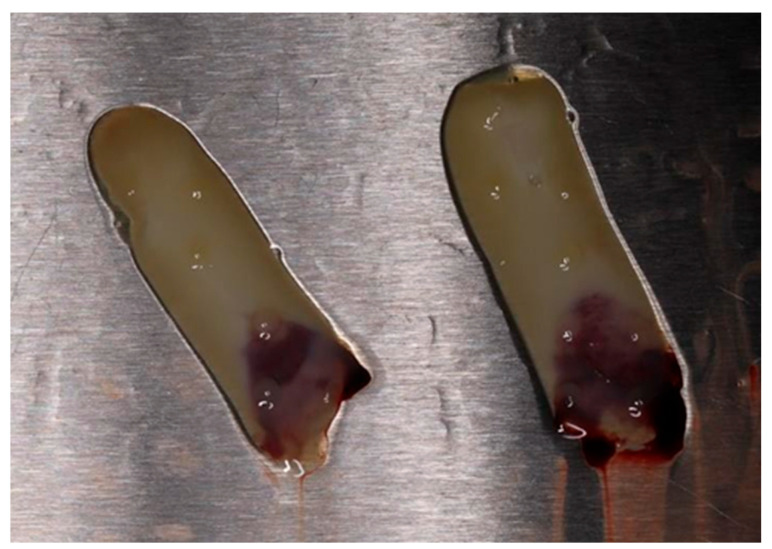
Advanced platelet-rich fibrin (A-PRF) membranes obtained after pressure applied onto buffy coat of centrifuged blood.

Venous blood was collected from each patient from the study group using sterile vacuum tubes (Matrix Choukroun) without additive. Immediately (in maximum 90 s), the tubes with whole blood (10 mL in each) were centrifuged by Duo Quattro (Choukroun PRFTM System) at 1300 rpm for 14 min. After centrifugation, A-PRF gel represented as the buffy coat in the middle layer was carefully isolated from the red blood cell clots. Half of the prepared A-PRF was pressurized to remove the liquid components to make A-PRF membrane for later use (Figure 1). The rest of the prepared A-PRF was used as such.

#### 2.3.3. Surgical Procedures

**Figure 2:**
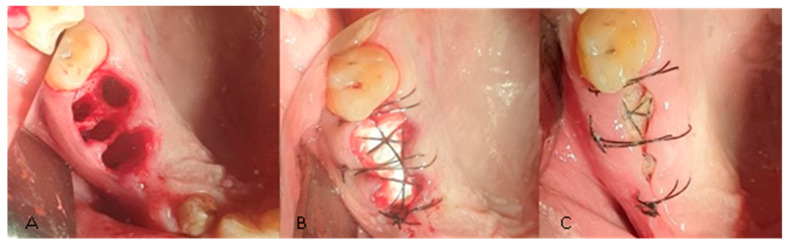
(**A**) Post-extractional alveoli. (**B**) Alveoli cu APRF and flattened collagen sponge and suture. (**C**) Surgical sites 7 days after the intervention.

Anesthesia was obtained by local infiltration with 4% articaine and 1:100,000 adrenaline (Ubistesin^TM^ Forte, 3M ESPE, Neuss, Germany). The affected tooth was extracted by atraumatic method, with root separation, using periotomes and residual root extractor. After removing the affected tooth, the periapical lesion was curetted by using bone curettes and the surgical area was then rinsed with physiological saline. For the control group, the post-extraction sockets were sutured immediately with non-absorbable polyamide 4-0 thread (Supramid, SMI sutures, Sankt Vith, Belgium). In the study group, all the post-extraction sockets were filled with A-PRF membranes both pressed and unpressed, then covered with a flattened collagen sponge (Collacone, Botiss Biomaterials, Berlin, Germany) which was fitted and sutured at the recipient site with a nonabsorbable 4-0 polyamide thread (Supramid, SMI sutures, Sankt Vith, Belgium) (Figure 2). No primary closure was attempted and no bone graft was used.

#### 2.3.4. Postoperative Protocol

All subjects received detailed verbal and written postoperative instructions and were prescribed 0.2% chlorhexidine mouthwash two times a day for 2 weeks, Ketonal Duo 150 mg once a day for 5 days, and to continue the antibiotic therapy started preoperative. The sutures were removed 14 days postoperatively and the healing was evaluated. After 3 months, the patients were recalled for CBCT evaluation to plan implantoprosthetic treatment.

### 2.4. CBCT Evaluation

CBCTs were performed using Carestream CS 8100 3D (Carestream Dental LLC, Atlanta, GA, USA), and interpretation was performed using Carestream 3D Imaging Software, 3D Suite 3.10.38 by the same operator. To evaluate the bone level lost after the extraction, initial and final measurements were both performed in the buccal–lingual plane. In order to have some clear measurements, the maxillary sinus and the mandibular canal were established as fixed reference points. The radiopaque lines of the buccal and lingual corticals were considered the alveolar walls.

**Figure 3:**
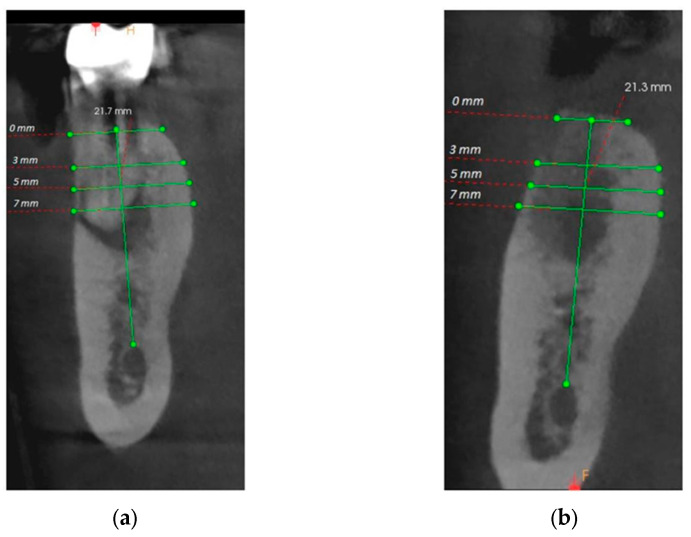
(**a**) Initial horizontal and vertical measurements; (**b**) Final horizontal and vertical measurements.

For the initial vertical dimension, the distance from the fixed points to the edge of the alveolus was measured by drawing the bisector that divides the alveolus into a buccal and a lingual portion, and for the final vertical dimension, the distance from the same fixed points to the top of the edentulous ridge was measured (Figure 3). To record the horizontal dimension, both initial and final, the buccal–lingual distance was measured in 4 different points, at 0, 3, 5, and 7 mm of height, compared to the top of the ridge, drawing lines that joined the two bone corticals.

### 2.5. Statistical Analysis

This study aimed to present the bone changes produced after ARP compared to spontaneous healing by measuring the vertical and horizontal dimensions of the bone, as is presented above in “CBCT evaluation”.

The data collected within this study were analyzed using Statistical Package for Social Sciences (SPSS), version 26 (IBM Corp., Armonk, NY, USA). Continuous variables were presented as mean ± standard deviation (SD) or medians. The bone changes produced after ARP, measuring the vertical and horizontal dimensions of the bone, were processed using the following tests: the Shapiro–Wilk test for normality distribution and the Levene test for homogeneity of variances. Baseline and follow-up measurements were analyzed using the paired samples *t*-test and Wilcoxon signed-rank test, respectively (when the distribution of differences between measurements before and after ARP was not normally distributed). The differences between baseline and follow-up values were analyzed using the independent samples *t*-test and Mann–Whitney U for group distributions. The threshold of 5% was chosen as statistically significant; therefore, the value *p* < 0.05 was used to determine the results of the statistical tests.

## 3. Results

**Figure 4:**
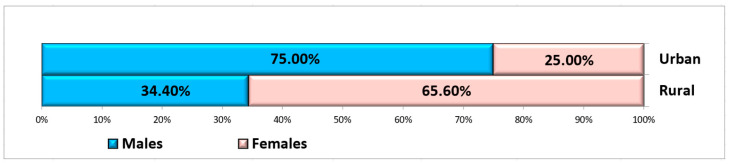
Distribution of subjects according to gender and residence area.

In this study, only non-smoking, systemically healthy patients who presented non-restorable teeth in the maxillary and mandibular molar areas indicated for extraction were included. A total of 84 sites were selected from 84 patients who met the inclusion criteria. The patients were divided into two groups: the study group (42 patients) and the control group (42 patients). Patients were both men and women, aged between 22 and 65 years (mean: 47.91 ± 11.45 years) and who came from both rural and urban areas (Figure 4). Details of the participants are shown in Table 1.

### 3.1. Baseline Data

**Figure 5:**
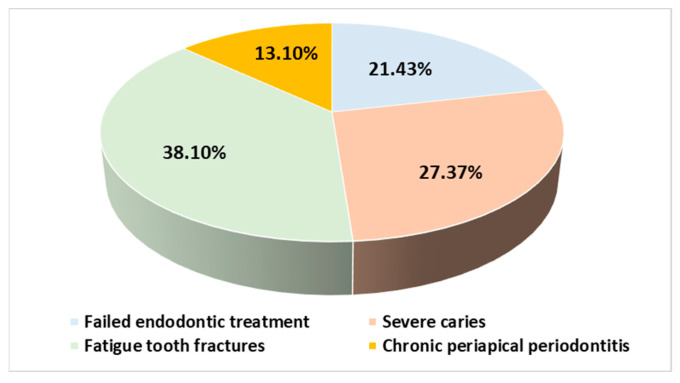
Distribution of subjects according to extraction etiology.

**Figure 6:**
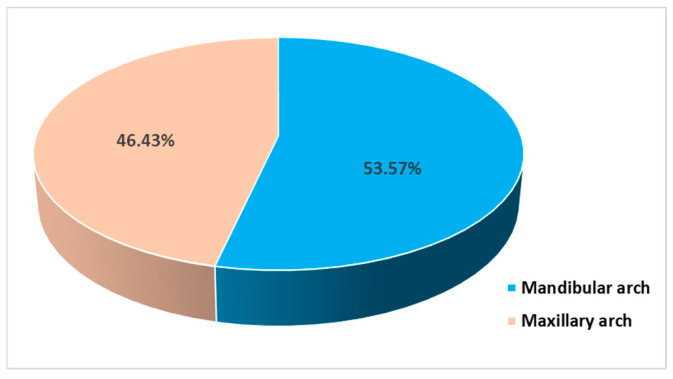
Distribution of subjects in relation to the extraction sites.

All participants completed the surgical protocols. The causes of the extractions were mainly fatigue tooth fractures (45.72%), severe caries (28.58%), failed endodontic treatments (21.42%), and chronic periapical periodontitis (4.28%) (Figure 5). The extractions were performed in the molar areas: 32 (45.72%) in the maxilla and 38 (54.28%) in the mandible (Figure 6). There were more mandibular teeth in the A-PRF group, and more maxillary teeth in the control group, but the differences were not statistically significant (χ^2^(1) = 1.497, *p* = 0.274). The details are presented in Table 2.

A total of 42 patients were included in the study group, of which 28 were men and 14 were women. There were 14 patients from rural areas and 28 from urban areas, aged between 22 and 65 years (mean: 45.90 ± 12.18 years) (Table 1). In this study group, there were 42 extractions performed: 25 (59.52% from all patients with A-PRF) on the mandible (13 first molars and 12 s molars) and 17 (40.48% from all patients with A-PRF) on the maxilla (11 first molars and 6 s molars). The main causes were failed endodontic treatment (14; 33.34% from all patients with A-PRF), severe caries (12; 28.57%), fatigue tooth fractures (5; 11.90%) and chronic periapical periodontitis (11; 26.19%) (Table 2). All post-extraction alveoli were preserved using A-PRF and flattened collagen sponge.

The control group included 42 patients, of which 22 men and 20 women. There were 17 patients from rural areas and 24 from urban areas, aged between 28 and 62 years (mean: 49.90 ± 10.42 years) (Table 1). In the case of the control group, 42 extractions were performed: 20 (47.62% of all control extractions) on the mandible (13 first molars and 7 s molars) and 22 (52.38%) on the maxilla (12 first molars and 10 s molars). The main causes were fatigue tooth fracture (27; 64.29% of all control extractions), severe caries (11; 26.19%) and failed endodontic treatment (4; 9.52%) (Table 2). In these patients, healing occurred spontaneously, without adding any material to the post-extraction alveoli.

### 3.2. Clinical Outcomes

All sockets healed uneventfully without signs of inflammation or other complications, and adverse reactions such as wound dehiscence and acute infection during the clinical healing period and the pain was moderate.

### 3.3. CBCT Analysis

**Figure 7:**
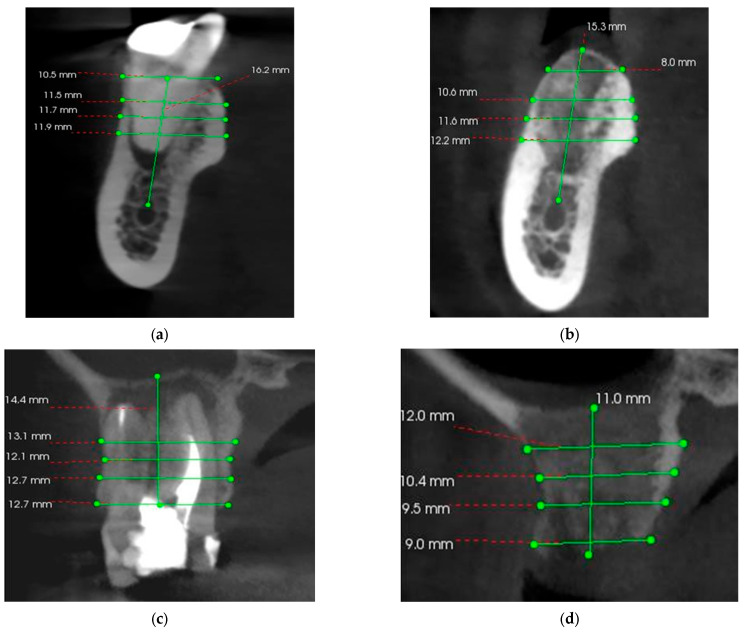
(**a**) Pre-operative CBCT at the level of the left mandibular first molar (36); (**b**) 3 months postoperative CBCT at the level of the left mandibular first molar (36); (**c**) Pre-operative CBCT at the level of the right maxillary first molar (16); (**d**) 3 months postoperative CBCT at the level of the right maxillary first molar (16).

CBCT analyzes were performed before the intervention and 3 months after the intervention in order to evaluate the possibility of implant treatment. The precise comparison of pre- and post-intervention data was made by choosing stable anatomical formations as reference points. For the maxilla, the reference point was the maxillary sinus, and for the mandible, it was the mandibular canal (Figure 7).

### 3.4. A-PRF/Control Subgroups Analysis

**Figure 8:**
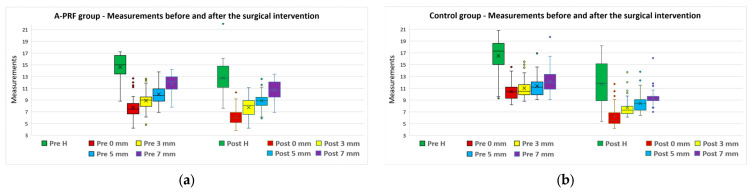
Measurements before (“Pre” values) and after (“Post” values) the surgical intervention for (**a**) A-PRF group and (**b**) control group.

After the initial CBCT analysis, the overall mean bone height was 15.55 ± 3.05 mm and the mean horizontal dimensions were as follows: at 0 mm, from 9.06 ± 2.21 mm, at 3 mm, from 9.94 ± 1.96 mm, at 5 mm, from 10.65 ± 1.78 mm, and at 7 mm, from 11.94 ± 1.90 mm. The measurements performed on the final CBCT showed loss of bone volume both vertically and horizontally, for both A-PRF and control groups (Figure 8a,b).

The differences between the values measured before and after ARP (pre–post differences) were computed for all five parameters, for both A-PRF and control group (Table 3). The percentage variation in measured values was defined as the ratio between the differences measured before and after ARP, and the initial value measured before ARP.

For A-PRF group, the differences were normally distributed only for heights; therefore, the variation of this parameter was analyzed based on a paired samples *t*-test, to determine whether there were statistically significant mean differences between the values measured before and after ARP. One outlier was detected that was less than 1.5 box-lengths from the edge of the box in a boxplot. Inspection of this value did not reveal it to be extreme and it was kept in the subsequent analysis. The normal distribution was assessed by the Shapiro–Wilk test (*p* = 0.275). For heights, the values measured after ARP expressed a statistically significant decrease compared to the values measured before ARP (t(41) = 12.62, *p* < 0.0005). For all four widths, the pre–post differences were not normally distributed, so the variation of this parameter was analyzed based on a Wilcoxon signed-rank test. There was a statistically significant median decrease for all width measurements: 0 mm (decrease from 7.45 to 5.90, z = −5.649, *p* < 0.0005), 3 mm (decrease from 9.00 to 8.10, z = −5.652, *p* < 0.0005), 5 mm (decrease from 9.80 to 8.90, z = −5.652, *p* < 0.0005), and 7 mm (decrease from 12.20 to 10.70, z = −5.585, *p* < 0.0005).

For the control group, for height, width at 0 mm, width at 3 mm, and width at 7 mm, the pre–post differences were normally distributed; therefore, the variation of these parameters was analyzed based on a paired samples *t*-test, to determine whether there were statistically significant mean differences between the two measurements. For all four parameters except widths at 0 mm, there were no outliers in the data, as assessed by inspection of a boxplot for values greater than 1.5 box-lengths from the edge of the box. For width at 0 mm, one outlier was detected that was less than 1.5 box-lengths from the edge of the box in a boxplot. Inspection of this value did not reveal it to be extreme and it was kept in the subsequent analysis. The normal distribution was assessed by the Shapiro–Wilk test (*p* = 0.324 for height, and *p* = 0.193, 0.213, and 0.606 for width at 0, 3, and 7 mm, respectively). For all four parameters, the values measured after the second measurement expressed a statistically significant decrease compared to the values measured before the intervention (*p* < 0.0005). For the width measured at 5 mm, the pre–post differences were not normally distributed, and the variation of this parameter was analyzed based on a Wilcoxon signed-rank test. There was a statistically significant median decrease in the width at 5 mm after the surgical procedure (8.40 mm) compared to the width at 5 mm before the intervention (11.10 mm), z = −5.647, *p* < 0.0005.

The differences between the values measured before and after the extraction (pre–post differences) for both study groups, A-PRF group and control group, are presented in Table 4. The percentage variation of the parameters was defined as the ratio between the differences measured for the A-PRF group, and the differences measured for the control group.

**Figure 9:**
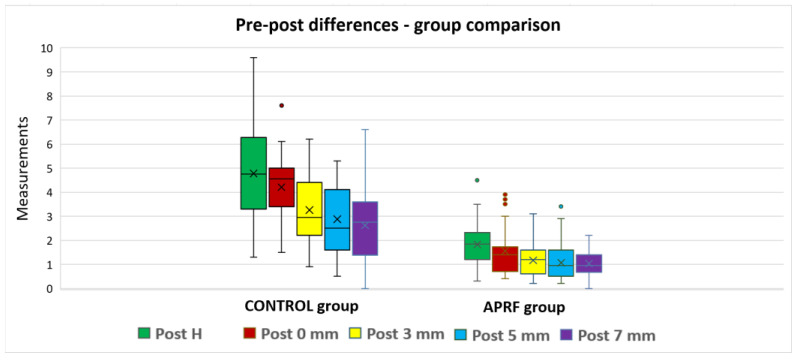
Measurements post-extraction: group comparison.

For all five parameters, the measurements were not normally distributed; therefore, the pre–post differences were analyzed based on a Mann–Whitney U test. The A-PRF group reported smaller average values for all parameters, compared to the control group, and the differences were statistically significant (*p* < 0.0005) (Table 4, Figure 9).

### 3.5. Maxillary/Mandible Subgroup Analysis

**Figure 10:**
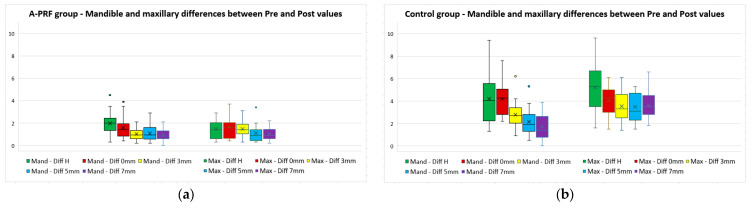
Maxillary and mandible differences between “Pre” and “Post” values for (**a**) A-PRF group and (**b**) control group.

The differences between the values measured before and after the extraction (pre–post differences), divided by maxillary/mandible, for all five parameters for both study groups, are presented in Table 5. The differences between maxillary and mandible measurements for both groups are also presented in Figure 10.

For the A-PRF maxillary subgroup, only for height, width 3 mm, and width at 7 mm, the differences were normally distributed; therefore, the variation of these parameters was analyzed based on a paired samples *t*-test, to determine whether there were statistically significant mean differences between the values measured before and after ARP. No outlier was detected. The normal distribution was assessed by the Shapiro–Wilk test, (for height, *p* = 0.435, for width at 3 mm, *p* = 0.452, and for width at 7 mm, *p* = 0.372). For all three parameters, the values measured after ARP expressed a statistically significant decrease compared to the values measured before ARP (for height, t(16) = 8.197, *p* < 0.0005; for width at 3 mm, t(16) = 9.561, *p* < 0.0005; for width at 7 mm, t(16) = 7.908, *p* < 0.0005). For width at 0 mm and 5 mm, the pre–post differences were not normally distributed, so the variation of these parameters was analyzed based on a Wilcoxon signed-rank test. There was a statistically significant median decrease for all width measurements: 0 mm (decrease from 8.10 to 6.60, z = −3.625, *p* < 0.0005), and 5 mm (decrease from 9.30 to 8.70, z = −3.652, *p* < 0.0005).

For the A-PRF mandible subgroup, only for height and width at 7 mm, the differences were normally distributed; therefore, the variation of these parameters was analyzed based on a paired samples *t*-test, to determine whether there were statistically significant mean differences between the values measured before and after ARP. No outlier was detected. The normal distribution was assessed by the Shapiro–Wilk test, (for height, *p* = 0.539, and for width at 7 mm, *p* = 0.385). For both parameters, the values measured after ARP expressed a statistically significant decrease compared to the values measured before ARP (for height, t(24) = 10.198, *p* < 0.0005; for width at 7 mm, t(24) = 9.773, *p* < 0.0005). For width at 0 mm, 3 mm, and 5 mm, the pre–post differences were not normally distributed, so the variation of these parameters was analyzed based on a Wilcoxon signed-rank test. There was a statistically significant median decrease for all width measurements: 0 mm (decrease from 8.10 to 6.60, z = −4.375, *p* < 0.0005), 3 mm (decrease from 8.90 to 7.20, z = −4.385, *p* < 0.0005), and 5 mm (decrease from 9.30 to 8.70, z = −4.374, *p* < 0.0005).

For the control maxillary subgroup, for all five parameters, the pre–post differences were normally distributed, as assessed by the Shapiro–Wilk test, (for height, *p* = 0.608, for width at 0 mm, *p* = 0.197, for width at 3 mm, *p* = 0.528, for width at 5 mm, *p* = 0.067, and for width at 7 mm, *p* = 0.360). The variation of these parameters was analyzed based on a paired samples *t*-test, to determine whether there were statistically significant mean differences between the values measured before and after ARP. One outlier was detected for width at 0 mm that was less than 1.5 box-lengths from the edge of the box in a boxplot. Inspection of this value did not reveal it to be extreme and it was kept in the subsequent analysis. For all five parameters, the values measured after ARP expressed a statistically significant decrease compared to the values measured before ARP (for height, t(21) = 13.126, *p* < 0.0005; for width at 0 mm, t(21) = 16.692, *p* < 0.0005; for width at 3 mm, t(21) = 13.757, *p* < 0.0005; for width at 5 mm, t(21) = 14.247, *p* < 0.0005; for width at 7 mm, t(21) = 14.921, *p* < 0.0005).

For the control mandible subgroup, only for height and width at 0 mm and at 7 mm, the differences were normally distributed; therefore, the variation of these parameters was analyzed based on a paired samples *t*-test, to determine whether there were statistically significant mean differences between the values measured before and after ARP. For each variable, no outliers were detected. The normal distribution was assessed by the Shapiro–Wilk test, (for height, *p* = 0.162, for width at 0 mm, *p* = 0.270, and for width at 7 mm, *p* = 0.093). For all three parameters, the values measured after ARP expressed a statistically significant decrease compared to the values measured before ARP (for height, t(19) = 9.068, *p* < 0.0005; for width at 0 mm, t(19) = 14.122, *p* < 0.0005; for width at 7 mm, t(19) = 6.052, *p* < 0.0005). For width at 3 mm and 5 mm, the pre–post differences were not normally distributed, so the variation of these parameters was analyzed based on a Wilcoxon signed-rank test. There was a statistically significant median decrease for both width measurements: 3 mm (decrease from 9.90 to 7.30, z = −3.925, *p* < 0.0005), and 5 mm (decrease from 9.85 to 8.40, z = −3.922, *p* < 0.0005).

The differences between maxilla and mandible values for each group, A-PRF and control, are presented in Table 6.

For the A-PRF group, only for height and width at 7 mm, the differences were normally distributed; therefore, the variation of these parameters was analyzed based on an independent samples *t*-test. No outlier was detected. The normal distribution was assessed by the Shapiro–Wilk test. For height, the mean differences were higher in mandible compared to the maxilla, but without a statistical significance (*p* = 0.074). For width at 7 mm, the differences measured in maxilla were higher than in mandible, also without a statistical significance (*p* = 0.263). For width at 0 mm, 3 mm, and 5 mm, the differences between maxilla and mandible were not normally distributed, so the variation of these parameters was analyzed based on a Mann–Whitney U test. There was a statistically significant difference only for width at 3 mm (*p* = 0.013).

For the control group, only for height, width at 0 mm, and width at 7 mm, the differences were normally distributed; therefore, the variation of these parameters was analyzed based on an independent samples *t*-test. No outlier was detected. The normal distribution was assessed by the Shapiro–Wilk test. For height and width at 7 mm, the mean differences were higher in maxilla compared to the mandible, and they were statistically significantly different (*p* < 0.05). For width at 3 mm and 5 mm, the differences between maxilla and mandible were not normally distributed, so the variation of these parameters was analyzed based on a Mann–Whitney U test. The resorption of the ridge was statistically significantly higher in maxilla compared to mandible (*p* < 0.05).

For both maxillary and mandible measurements, for all five parameters, the differences were statistically significantly higher in the control group compared to the A-PRF group (*p* < 0.05) (Table 7).

## 4. Discussion

Autologous products such as platelet-rich plasma (PRP) and platelet-rich fibrin (PRF) have an important role in tissue healing and are therefore frequently used in dentistry and especially in surgery [54,55]. Through this retrospective study, the vertical and horizontal bone changes in first and second molars that occurred following tooth extraction and consecutive alveolar ridge preservation using A-PRF compared to tooth extraction and spontaneous healing were evaluated. Analyzing the CBCT, the bone dimensions before the intervention and 3 months after it were compared. Dental extractions were performed in the maxillary and mandibular molar areas. Most of the patients were male, and the highest number of extractions was performed on the mandible. The same surgical protocol was followed for all patients, except the ridge-preservation technique, and no signs of inflammation or other complications and adverse reactions were observed during the follow-up period. Instead, the reduction in bone volume was found both vertically and horizontally, but significantly smaller in the study group in which ARP was performed than in the control group with spontaneous healing. Bone resorption in the maxilla occurred with the narrowing of the edentulous ridge, compared to the mandible, where the shape of the ridge was better maintained after extraction. The height of the edentulous ridge did not differ much in terms of variation in the two groups, in the maxilla compared to the mandible. Differences between the maxilla and the mandible were found only in terms of the thickness of the edentulous ridge (H = 5, H = 7) only in the control group. The maxillary–mandibular differences in the A-PRF group were not statistically significant. There was a statistically significant difference only for width at 3 mm. The significance of this phenomenon resides in a healing/resorption process similar to the maxilla/mandible when A-PRF is used post-extraction [16,28].

The results were similar to other studies in the literature. For example, in the study conducted by Araujo et. al., the results showed a reduction in bone volume of 25% in the group with spontaneous healing and only 3% for the group in which the alveolar ridge was preserved [1]. Also, in other studies in which the same comparisons were made, the results were favorable for the preservation of the alveolar ridge [8,18,22,35,46,47,56,57]. The studies that analyzed the results of implant therapy had better outcomes for the preserved ridges than spontaneously healed alveoli [27,36,58]. In the present study, only the molar areas (first and second maxillary and mandibular molars) were included in order to homogenize the characteristics of the study sample and to minimize as much as possible the differences in size and morphology of the post-extraction alveoli on the results obtained.

Alveolar bone resorption that occurs immediately after tooth extraction is the main problem that affects the outcome of the implant prosthetic treatment and subsequently, the stomatognathic system functions [8,9,59]. To be able to restore functionality and to achieve long-term success with implant therapy, it is necessary to have sufficient bone volume [27,58]. Therefore, the alveolar bone must be carefully managed during tooth extraction and after it in order to promote bone preservation [2,4,7,8,59,60]. Minimally invasive surgical procedures can lead to better results in terms of the bone remodeling process because tissue injuries have an influence on the speed and quality of alveolar bone healing [61,62]. In this study, extractions were performed with minimal trauma. Also, the integrity of the periosteum is very important because this can be the key to minimal bone resorption and to improving re-ossification [61,62]. Therefore, bone regeneration depends on the material used to alveolar ridge preservation and equally on the integrity of the periosteum [61,62]. Comparing natural healing with alveolar ridge preservation using concentrate growth factors with bone substitute, ARP had significant advantages in preserving bone size and reducing bone resorption [2,28,55,60]. The morphology of the post-extraction alveoli often requires reconstructive surgical procedures before or at the time of implant insertion, thus ensuring adequate bone volume and long-term stability of the implants [5,27].

Numerous studies have been conducted with regard to alveolar healing processes after tooth extraction and ARP procedure [2,6,8,18,59,63,64]. In general, the techniques for alveolar ridge preservation are based on the introduction of materials such as bone grafts into the post-extraction alveolus, and then sealing it with a membrane [2,4]. The effectiveness of these interventions has been evaluated in several studies, and it has been well documented that ARP procedures result in a significant reduction in bone resorption compared with management without ARP [2,4,6,59,64]. In the study carried out by El-Sioufi et al., the results obtained after alveolar ridge preservation with different materials were compared. Their conclusions showed a significantly lower bone resorption in the case of bone graft materials than in the case where only free gingival graft was performed [64]. In another study, Azangookhiavi et al. [45] compared the results of applying FDBA and PRF for the preservation of the alveolar ridge in post-extraction alveoli. The reduction in ridge width was 1.1–2.0 mm in the PRF group and 0.5–1.4 mm in the FDBA group, and the reduction in ridge height was 0.1–0.7 mm in the PRF group and 0–0.2 mm in the FDBA group. Without ridge preservation, the mean changes in ridge width and height at six months were 3.87 mm and 1.67 mm, respectively. Improved three-dimensional (3D) structure of bone and positive effects on bone quality can be justified by the presence of growth factors trapped in PRF and their effect on recently formed bone trabeculae in the socket [45].

PRF, a second-generation platelet concentrate collected on a single fibrin membrane, contains all the constituents favorable to healing and stimulates human osteoblastic proliferation and has a positive effect on neoangiogenesis [54,55,56]. In a membrane form, it can be used as fibrin bandage, serving as a matrix to accelerate the healing of wound edges [54,55,57]. It is a relatively cheap technique that is performed quickly and easily. Following the filling of the post-extraction alveolus with the PRF clot, neovascularization is established, and an epithelial covering develops. Although there is potential for infection and inflammation of the extraction socket, rapid wound healing occurs without pain or other complications [54,55,56,65,66]. The next generation of PRF, advanced platelet-rich fibrin (A-PRF), is obtained in low-speed centrifugation, and is considered a new autologous material that has numerous benefits in implant dentistry [58]. A-PRF is an important source of growth factors that accelerate the healing stages, thus shortening the rehabilitation time of an edentulous space. Accelerated healing in post-extraction sockets was studied in an article comparing healing in grafted premolar sockets with and without A-PRF [67].

A-PRF stimulates the clinically observed soft tissue healing process when applied to post-extraction alveoli. In addition, it appears to reduce resorption of the alveolar ridge following tooth extractions and to positively influence alveolar healing [42,46,47,48]. A-PRF helped increase bone density, epithelial healing, and control postoperative pain and swelling after tooth extraction [46]. In the study carried out by Makki et al. comparing the use of A-PRF with L-PRF in post-extraction alveoli, it was shown that the use of A-PRF significantly reduced postoperative pain and improved early soft tissue healing [66]. The same results were obtained in other studies in the literature [67,68,69,70,71]. A-PRF is a generation of PRF in solid form, which is safe and more clinically effective, approved in preclinical and clinical studies, compared to other generations of PRF. A-PRF, compared to other platelet concentrates, showed a significant increase in the release of growth factors [47]. Also, in other studies, the results showed that the group in which ARP was performed with the help of A-PRF experienced a significantly smaller reduction in ridge height compared to the control groups [41,42,46,47,48].

The results of this study were similar to the results of the study conducted by Srinivas et al., which showed that PRF provided appreciable wound healing and bone regeneration compared to sites where spontaneous healing was resorted to without PRF [56]. In the study by Clark et al., better results were obtained in the case of patients who underwent ARP with A-PRF alone or in combination with FDBA, than in the case of spontaneous healing. They validated A-PRF as the desired material for post-extraction alveolar ridge preservation, showing that A-PRF led to the most vital bone formation, the main benefit of this biomaterial being the dense fibrin structure [39]. In the study by Gowda et al., a significant decrease in BLRW, MBH, and MPH/MLH was obtained in the A-PRF+ group, while significantly larger socket sizes were shown in the PDDM control group. The reduction in bone resorption was greater in the A-PRF+ group compared to the PDDM group for all clinical parameters, and the difference between the two groups was also statistically significant (*p* < 0.05) [72]. In the study conducted by Hauser et al., it has been shown that an invasive surgical procedure such as buccal extraction flap and primary closure seems to neutralize the advantages of PRF [63]. Thus, in this study, the primary closure was not attempted. With A-PRF as a graft material, the healing process is fast and does not have a reaction associated with a foreign body, being an autologous material. Lekovic et al. obtained a change in alveolar width and height and recorded a height loss of 0.38 mm (11.59%) after 4–6 months of healing [73]. In their study, Agarwal et al. showed a vertical bone reduction of almost 2 mm [74], and Srinivas et al. showed a reduction in alveolar bone of 0.06 ± 0.25 mm where the mean height was not significantly reduced at 3 months after treatment follow-up with the use of PRF [56].

On the other hand, the results of the present study were different from some studies in the literature, such as the study conducted by Pereira et al. [75], which shows that the use of A-PRF+ does not demonstrate a clinical advantage in post-extraction alveolar repair of third upper molars. Lahham et al. also reported less efficiency on non-molars for A-PRF+ compared to recurrent applications of concentrated PRF, over a period of two months [47].

Considering that the literature reports failures of bone grafts in the post-extraction alveoli after long periods of time after implantation [76], the use of biological solutions such as A-PRF to preserve the size of the post-extraction alveolus is a much better alternative, with the material introduced into the alveolus being autologous, without risk of adverse reactions, easy to process, and with significantly lower costs. A-PRF used for post-extraction socket preservation significantly reduces bone resorption and helps the bone remodeling process. However, further studies with histological analysis and a longer follow-up period are needed to confirm the obtained results.

Limitations of the study are represented by the reduced number of patients and the fact that their selection could have been more randomized; however, these were the patients available within the study timeframe.

## 5. Conclusions

A-PRF used as grafting material of the post-extraction alveolus improved healing, with post-extraction bone resorption being significantly lower compared to the control group in which simple extraction was performed. Bone resorption was observed in all cases, both vertically and horizontally, more in the maxilla than in the mandible. Because all patients underwent atraumatic extractions, no complications or adverse reactions were reported during the follow-up period.

## Figures and Tables

**Table 1:** Sample demographics.

Study Variable	A-PRF Group	Control Group	TOTAL	*p*
Patients (n/%)	42 (50.00%)	42 (50.00%)	84 (100%)	-
Age (years)—median	48	52		0.094
Gender				
Female (n/%)	14 (41.20%)	20 (58.80%)	34 (100%)	0.182
Male (n/%)	28 (56.00%)	22 (44.00%)	50 (100%)	
Residence				
Urban area (n/%)	28 (53.80%)	24 (46.20%)	52 (100%)	0.369
Rural area (n/%)	14 (43.80%)	18 (56.20%)	31 (100%)	

**Table 2:** Dental extraction data.

Variables		A-PRF Group	Control Group	No. of Sites
Extracted teeth		42 (50.00%)	42 (50.00%)	84 (100%)
Location	Mandibular teeth	25 (55.56%)	20 (44.40%)	45 (100%)
	Maxillary teeth	17 (43.60%)	22 (56.40%)	39 (100%)
Extraction causes	Failed endodontic treatments	14 (77.77%)	4 (22.23%)	18 (100%)
	Severe caries	12 (52.17%)	11 (47.83%)	23 (100%)
	Fatigue tooth fractures	5 (15.62%)	27 (84.38%)	32 (100%)
	Chronic periapical periodontitis	11 (100%)	0 (0.0%)	11 (100%)

**Table 3:** Dimensional changes (expressed in mm) between baseline and final follow-up. Main average and standard deviation (SD).

Parameter	Baseline (mm)	Follow-Up (mm)	Mean Difference (mm)	Mean Percentage Variation (%)	*p*
A-PRF group					
Height	14.60 ± 2.68	12.77 ± 2.86	1.83 ± 0.94	12.91%	˂0.0005 ^1^
Width at 0 mm	7.73 ± 1.99	6.17 ± 1.47	1.56 ± 0.92	19.35%	˂0.0005 ^2^
Width at 3 mm	8.90 ± 1.70	7.74 ± 1.57	1.17 ± 0.62	13.15%	˂0.0005 ^2^
Width at 5 mm	9.98 ± 1.61	8.90 ± 1.44	1.07 ± 0.70	10.59%	˂0.0005 ^2^
Width at 7 mm	11.80 ± 1.67	10.76 ± 1.61	1.03 ± 0.54	8.74%	˂0.0005 ^2^
Control group					
Height	16.50 ± 3.14	11.73 ± 3.70	4.78 ± 2.06	30.05%	˂0.0005 ^1^
Width at 0 mm	10.40 ± 1.52	6.20 ± 1.77	4.20 ± 1.24	40.79%	˂0.0005 ^1^
Width at 3 mm	10.99 ± 1.62	7.73 ± 1.64	3.26 ± 1.26	29.56%	˂0.0005 ^1^
Width at 5 mm	11.32 ± 1.71	8.45 ± 1.56	2.88 ± 1.44	24.87%	˂0.0005 ^2^
Width at 7 mm	12.09 ± 2.12	9.47 ± 1.56	2.62 ± 1.52	20.78%	˂0.0005 ^1^

^1^ Paired samples *t*-test; ^2^ Wilcoxon signed-rank test.

**Table 4:** Differences expressed in mm between baseline and final follow-up dimensional changes. Main average and standard deviation (SD).

Parameter	A-PRF Group (Median)	Control Group (Median)	Percentage Variation (%)	U/z ^1^	*p* ^1^
Height	1.85	4.75	38.58%	1597.00/6.401	˂0.0005
Width at 0 mm	1.40	4.55	36.88%	1676.00/7.108	˂0.0005
Width at 3 mm	1.20	2.95	35.56%	1677.00/7.109	˂0.0005
Width at 5 mm	0.95	2.50	36.61%	1552.50/6.004	˂0.0005
Width at 7 mm	0.95	2.75	38.73%	1448.50/5.072	˂0.0005

^1^ Mann–Whitney U test.

**Table 5:** Dimensional changes (expressed in mm) between baseline and final follow-up. Main average and standard deviation (SD).

Parameter	Baseline (mm)	Follow-Up (mm)	Mean Percentage Variation (%)	*p*
A-PRF group—Maxilla				
Height	13.61 ± 2.92	12.09 ± 2.84	11.40%	<0.0005 ^1^
Width at 0 mm	8.21 ± 2.04	6.59 ± 1.48	18.84%	<0.0005 ^2^
Width at 3 mm	8.85 ± 1.71	7.38 ± 1.41	16.46%	<0.0005 ^2^
Width at 5 mm	9.75 ± 1.55	8.61 ± 1.16	11.28%	<0.0005 ^2^
Width at 7 mm	11.63 ± 1.63	10.48 ± 1.48	9.76%	<0.0005 ^1^
A-PRF group—Mandible				
Height	15.28 ± 2.32	13.24 ± 2.83	13.94%	<0.0005 ^1^
Width at 0 mm	7.40 ± 1.93	5.88 ± 1.42	19.70%	<0.0005 ^2^
Width at 3 mm	8.94 ± 1.73	7.98 ± 1.66	10.90%	<0.0005 ^1^
Width at 5 mm	10.13 ± 1.66	9.11 ± 1.60	10.13%	<0.0005 ^2^
Width at 7 mm	11.91 ± 1.73	10.96 ± 1.69	8.04%	<0.0005 ^1^
Control group—Maxilla				
Height	14.54 ± 3.04	9.12 ± 2.72	37.56%	<0.0005 ^1^
Width at 0 mm	11.12 ± 1.46	6.99 ± 2.14	38.15%	<0.0005 ^1^
Width at 3 mm	11.84 ± 1.63	8.15 ± 2.17	31.75%	<0.0005 ^1^
Width at 5 mm	12.26 ± 1.62	8.59 ± 2.09	30.42%	<0.0005 ^1^
Width at 7 mm	13.23 ± 2.01	9.64 ± 1.90	27.21%	<0.0005 ^1^
Control group—Mandible				
Height	18.67 ± 1.26	14.60 ± 2.20	21.80%	<0.0005 ^1^
Width at 0 mm	9.62 ± 1.16	5.33 ± 0.48	43.70%	<0.0005 ^1^
Width at 3 mm	10.05 ± 0.99	7.26 ± 0.41	27.14%	<0.0005 ^1^
Width at 5 mm	10.30 ± 1.11	8.30 ± 0.63	18.76%	<0.0005 ^1^
Width at 7 mm	10.84 ± 1.43	9.28 ± 1.11	13.72%	<0.0005 ^1^

^1^ Paired samples *t*-test; ^2^ Wilcoxon signed-rank test.

**Table 6:** Differences expressed in mm between baseline and final follow-up dimensional changes, for maxilla and mandible, for both study groups. Main average and standard deviation (SD), and median.

Parameter	Maxilla (mm)	Mandible (mm)	*p*
A-PRF group			
Height	1.52 ± 0.76	2.04 ± 1.00	0.074 ^1^
Width at 0 mm	1.61 ± 0.96	1.52 ± 0.91	0.598 ^2^
Width at 3 mm	1.47 ± 0.63	0.96 ± 0.53	0.013 ^2^
Width at 5 mm	1.14 ± 0.80	1.02 ± 0.64	0.709 ^2^
Width at 7 mm	1.15 ± 0.60	0.96 ± 0.49	0.263 ^1^
Control group			
Height	5.41 ± 1.93	4.08 ± 2.01	0.034 ^1^
Width at 0 mm	4.13 ± 1.16	4.29 ± 1.36	0.696 ^1^
Width at 3 mm	3.68 ± 1.26	2.79 ± 1.11	0.016 ^2^
Width at 5 mm	3.67 ± 1.21	2.00 ± 1.16	<0.0005 ^2^
Width at 7 mm	3.59 ± 1.13	1.56 ± 1.15	˂0.0005 ^1^

^1^ Independent samples *t*-test. ^2^ Mann–Whitney U test.

**Table 7:** Differences expressed in mm between baseline and final follow-up dimensional changes—median values.

Parameter	A-PRF Group (Median)	Control Group (Median)	*p*
Maxilla			
Height	1.40	5.90	˂0.0005 ^1^
Width at 0 mm	1.60	4.35	˂0.0005 ^1^
Width at 3 mm	1.40	3.75	˂0.0005 ^1^
Width at 5 mm	1.00	3.85	˂0.0005 ^2^
Width at 7 mm	1.10	3.40	˂0.0005 ^1^
Mandible			
Height	2.20	3.70	˂0.0005 ^1^
Width at 0 mm	1.40	4.70	˂0.0005 ^2^
Width at 3 mm	0.80	2.70	˂0.0005 ^2^
Width at 5 mm	0.90	1.75	0.001 ^2^
Width at 7 mm	0.80	1.35	0.039 ^1^

^1^ Independent samples *t*-test. ^2^ Mann–Whitney U test.

## Data Availability

The authors declare that the data of this research are available from the corresponding authors upon reasonable request.
